# Genotype selection for phytochemical content and pharmacological activities in ethanol extracts of fifteen types of *Orthosiphon aristatus* (Blume) Miq. leaves using chemometric analysis

**DOI:** 10.1038/s41598-020-77991-2

**Published:** 2020-12-01

**Authors:** Irmanida Batubara, Komariah Komariah, Apong Sandrawati, Waras Nurcholis

**Affiliations:** 1 Tropical Biopharmaca Research Center, IPB University (Bogor Agricultural University), Bogor, Indonesia; 2 Department of Chemistry, Faculty of Mathematics and Natural Sciences, IPB University (Bogor Agricultural University), Bogor, Indonesia; 3 Department of Soil Science, Faculty of Agriculture, Sebelas Maret University, Surakarta, Indonesia; 4 Department of Soil Science and Land Resources, Faculty of Agriculture, Padjadjaran University, Bandung, Indonesia; 5 Department of Biochemistry, Faculty of Mathematics and Natural Sciences, IPB University (Bogor Agricultural University), Bogor, Indonesia

**Keywords:** Biochemistry, Chemical biology, Plant sciences

## Abstract

*Orthosiphon aristatus* (Blume) Miq. of the *Lamiaceae* family, called as kumis kucing in Indonesia, is a valuable medicinal plant for their pharmacological properties. The present study comprised of fifteen genotypes of *O. aristatus* was undertaken to evaluate the genotypes based on phytochemical content and pharmacological activities of leaves ethanol extract. Chemometric analysis (correlation and principal component analysis) was also used to investigate the genetic variability based on phytochemical content and pharmacological activities of *O. aristatus* genotypes. Results of phytochemical characterization showed that total phenolic ranged from 1.48 to 36.08 (maximum in A15) mg GAE/g DW, total flavonoid ranged from 0.10 to 3.07 (maximum in A15) mg QE/g DW, sinensetin ranged from 0.36 to 4.02 (maximum in A11) mg/g DW, and rosmarinic acid ranged 0.06 to 7.25 (maximum in A7) mg/g DW. Antioxidant activity was tested using DPPH and FRAP assay. Antioxidant results showed that DPPH ranged from 1.68 to 15.55 (maximum in A15) μmol TE/g DW and FRAP ranged from 0.07 to 1.60 (maximum in A1 and A7) μmol TE/g DW. The genotype A8 showed the highest cytotoxic activities against HeLa (66.25%) and MCF-7 (61.79%) cell lines. Maximum α-glucosidase inhibitory activity was recorded in genotype A2 with the value of 62.84%. The genotypes A1, A2, A7, A11, and A15 were identified as superior based on their phytochemicals content and pharmacological activities coupled with chemometric analysis. This finding is important for breeding studies and also the pharmaceutical perspective of *O. aristatus*.

## Introduction

*Orthosiphon aristatus* (Blume) Miq. known as kumis kucing in Indonesia belongs to the *Lamiaceae* family. It is a herb with many traditionally uses to treat several diseases such as bacterial and urinary tract infections, inflammation, rheumatism, influenza, angiogenesis, and jaundice^1–3^. *O. aristatus* is widely cultivated in tropical countries and Southeast Asia including Indonesia^4^. Asian and European people use the leaves of this plant as a herb tea that called as Java tea^4^. Several works related to pharmacological properties of *O. aristatus* have been carried out comprehensively, including antidiabetic^5^, anti-infective^6^, hypouricemic and diuretic ^7^, antiobesity and hypolipidemic^8^, antiosteoporotic^9^, antiangiogenic and anticancer^10,11^, antioxidant and protection of the intestine from oxidative stress^12,13^, osteoarthritis protection^14^, angiotensin I-converting enzyme and α-glucosidase inhibitory^15,16^. Phenolics, flavonoids, sinensetin, and rosmarinic acid are important constituents or components for pharmacological properties^17–19^. Therefore, the phytochemicals contents of *O. aristatus* is associated with their pharmacological activities. For this reason, it’s essential to select *O. aristatus* genotypes with high phytochemical levels and pharmacological activities, which can be used for a variety development through plant breeding programs.

The quality and yields of phytochemicals, leading to diversity of pharmacological activities in medicinal plants, can be influenced by environmental conditions and genotypes^20,21^. Therefore, in terms of the genotype selected under the particular environmental situation, the most appropriate varieties' development has become essential, which can increase their phytochemical and pharmacological activities. Plant varieties from a breeding program must have stability yield under various growing environmental conditions^22^. The assessment of genetic variability among various genotypes is the key requirement in the plant breeding program. Recently, breeders still use genetic and phenotypic performance to select important characters in the breeding program. Microsatellite markers have been used in genetic diversity analyses of twenty-eight samples of *O. aristatus* from Malaysia^23^. The diversity of morphological characters were identified in eighteen *O. aristatus* accessions from Indonesia^24^. Therefore, it is crucial to improve detailed comparative studies on plant chemicals' contents and their pharmacological activities of *O. aristatus*.

The present study was aimed to investigate the leaves phytochemical content and biological activities in ethanol extract of fifteen *O. aristatus* genotypes. Phytochemical analyses included the evaluation of phenolics, flavonoids, sinensetin, and rosmarinic acid, whereas the antioxidant, cytotoxicity and α-glucosidase inhibitory activities were performed for pharmacological activities. *O. aristatus* genotypes were grown under the same soil conditions and environment; therefore, the diversity of results was genetically reflected in the various genotypes which studied. *O. aristatus* genotypes were classified based on their phytochemical content and biological activities using correlation and principal component analysis (PCA). This result is significant in the identification of the elite genotypes for developing *O. aristatus* varieties at a commercial scale.

## Results

### Extraction yield

The results of ethanol extraction demonstrated the influence of genotypes on the extraction yield of *O. aristatus* leaves genotypes. The ethanol extraction yield of the studied genotypes varied from 1.73 to 16.91% DW (Table 1). The genotype A1 had the highest extraction yield, which not significantly different from A11 (16.64% DW) and A15 (16.67% DW) at *p* < 0.05, and the genotype A6 had the lowest.

**Table 1:** Extraction yield, TPC, TFC, SC, and RAC of fifteen *O. aristatus* genotype in ethanolic extracts of leaves.

Genotypes	EY	TPC	TFC	RAC	SC
	(% DW)	(mg GAE/g DW)	mg QE/g DW	(mg/g DW)	(mg/g DW)
A1	16.91a	15.27d	2.41b	1.18i	1.21e
A2	9.00e	5.76j	1.24f	2.67e	1.82d
A3	12.84c	5.28j	1.87d	4.75c	0.53fg
A4	15.35b	5.09j	1.99d	2.06g	0.73f
A5	15.25b	16.82cd	1.72c	3.84d	2.17c
A6	1.73h	1.48k	0.10i	0.06k	0.61fg
A7	14.98b	17.62c	0.84e	7.25a	1.45e
A8	13.34c	14.84e	1.72e	6.27b	0.36g
A9	10.75d	7.10i	0.89g	2.40ef	0.55fg
A10	11.53d	12.79f	1.13g	2.24f	0.87f
A11	16.64a	25.95b	2.13c	1.98g	4.02a
A12	9.11e	10.44e	0.38h	0.63j	0.88f
A13	7.86f	9.25g	0.41h	1.66h	0.20g
A14	5.34g	8.02h	0.76g	0.56j	0.40g
A15	16.67a	36.08a	3.07a	0.90i	3.31b

*DW* dried weight, *EY* extraction yield, *TPC* total phenolic content, *TFC* total flavonoids content, *SC* sinensetin content, *RAC* rosmarinic acid content; different letters in the same column represent statistically different results at p < 0.05.

### Phytochemical analysis

The results of the phytochemical analysis in ethanol extract of *O. aristatus* genotypes leaves are presented in Table 1. Total phenolic content (TPC), total flavonoid content (TFC), rosmarinic acid content (RAC), and sinensetin content (SC) have been evaluated in ethanol extract of 15 *O. aristatus* genotypes leaves. TPC varied significantly and ranged from 1.48 mg GAE/g DW to 36.08 mg GAE/g DW. The genotype A15 had the highest TPC, and the genotype A6 had lowest. TFC were observed statistically significant difference at p < 0.05, ranged from 0.10 mg QE/g DW to 3.07 mg QE/g DW among the *O. aristatus* genotypes. In this study, the maximum TFC was recorded with A15 and minimum was in A6. The highest RAC (7.25 mg/g DW) was found in genotype A7, meanwhile the lowest (0.06 mg/g DW) was observed in genotype A6. The SC ranged from 0.20 (A13) to 4.02 (A11) mg/g DW.

### Pharmacological activities

Antioxidant activity in the studied genotypes were measured using DPPH and FRAP assays (Table 2). In the DPPH assay, the antioxidant properties ranged between 1.68 (A6) to 15.55 (A15) μmol TE/g DW. DPPH value of A15 showed not significantly different with A1 (15.43 μmol TE/g DW) at p < 0.05. In FRAP assay, the genotype A1 and A7 showed maximum antioxidant properties with the same value of 1.60 mmol TE/g DW. In comparison, genotype A6 exhibited the lowest activity with a value of 0.07 mmol TE/g DW.

**Table 2:** Antioxidant activity, anticancer activity, and α-glucosidase inhibitory activity of fifteen *O. aristatus* genotype in ethanolic extracts of leaves.

Genotypes	DPPH	FRAP	HeLa	MCF-7	GIA
	(μmol TE/g DW)	(μmol TE/g DW)	(%)	(%)	(%)
A1	15.43a	1.60a	52.62c	36.70e-h	17.03i
A2	7.87ef	0.42h	57.49bc	57.02ab	62.84a
A3	12.37c	1.28c	52.43c	50.90bc	23.15gh
A4	14.28ab	0.82f	55.67bc	41.46def	15.41i
A5	13.75b	0.91e	53.88bc	58.56ab	6.28j
A6	1.68h	0.07i	50.76c	46.84cd	16.30i
A7	11.73c	1.60a	60.37ab	31.00gh	36.21e
A8	11.12d	1.36c	66.25a	61.79a	31.46f
A9	10.28d	0.83f	35.62e	21.96i	22.06h
A10	10.52d	1.19d	7.33f	35.70fgh	39.69d
A11	14.93a	1.50b	30.77e	44.49cde	43.95c
A12	8.83e	0.50g	34.66e	30.44h	52.64b
A13	7.25f	0.80f	43.30d	38.96d-g	25.15g
A14	5.07g	0.44h	28.85e	56.41ab	33.14f
A15	15.55a	1.32c	30.44e	56.57ab	43.24c

*DW* dried weight, *DPPH* 2,2-diphenyl-1-picrylhydrazyl, *FRAP* ferric reducing antioxidant power, *HeLa* anticancer activity of HeLa cancer cell line, *MCF-7* anticancer activity of MCF-7 cancer cell line, *GIA* α-glucosidase inhibitory activity; different letters in the same column represent statistically different results at p < 0.05.

Cytotoxic activities against HeLa and MCF-7 cell lines of 15 genotypes of *O. aristatus* extracts are exhibited in Table 2. Cytotoxic activity of *O. aristatus* genotypes against the HeLa cell line ranged from 7.33 to 66.25%. In the MCF-7 cell line, the cytotoxic activity of *O. aristatus* genotypes ranged from 21.96 to 61.79%. For both cell lines studied, genotype A8 showed the highest cytotoxic activity. The antiproliferative activity of genotype A8 against the HeLa cell line presented not significantly different from genotype A7 (60.37%). Meanwhile, in MCF-7 cell lines, cytotoxic activity of genotype A8 showed no difference with A2 (57.02%), A5 (58.56%), A14 (56.41%), and A15 (56.57%).

The *α-*glucosidase inhibition is used to select the *O. aristatus* varieties with the most antidiabetic potential. The 15 extracts of *O. aristatus* leaves from different genotypes had a different activity to inhibit *α-*glucosidase (Table 2). The inhibition activity was varied from 6.28% to 62.84%. The highest inhibition found in genotypes of A2, whereas the lowest detected in A5.

### Multivariate analysis

In this study, the correlation and principal component analysis (PCA) in *O. aristatus* studied genotypes were performed for multivariate data analysis.

**Figure 1:**
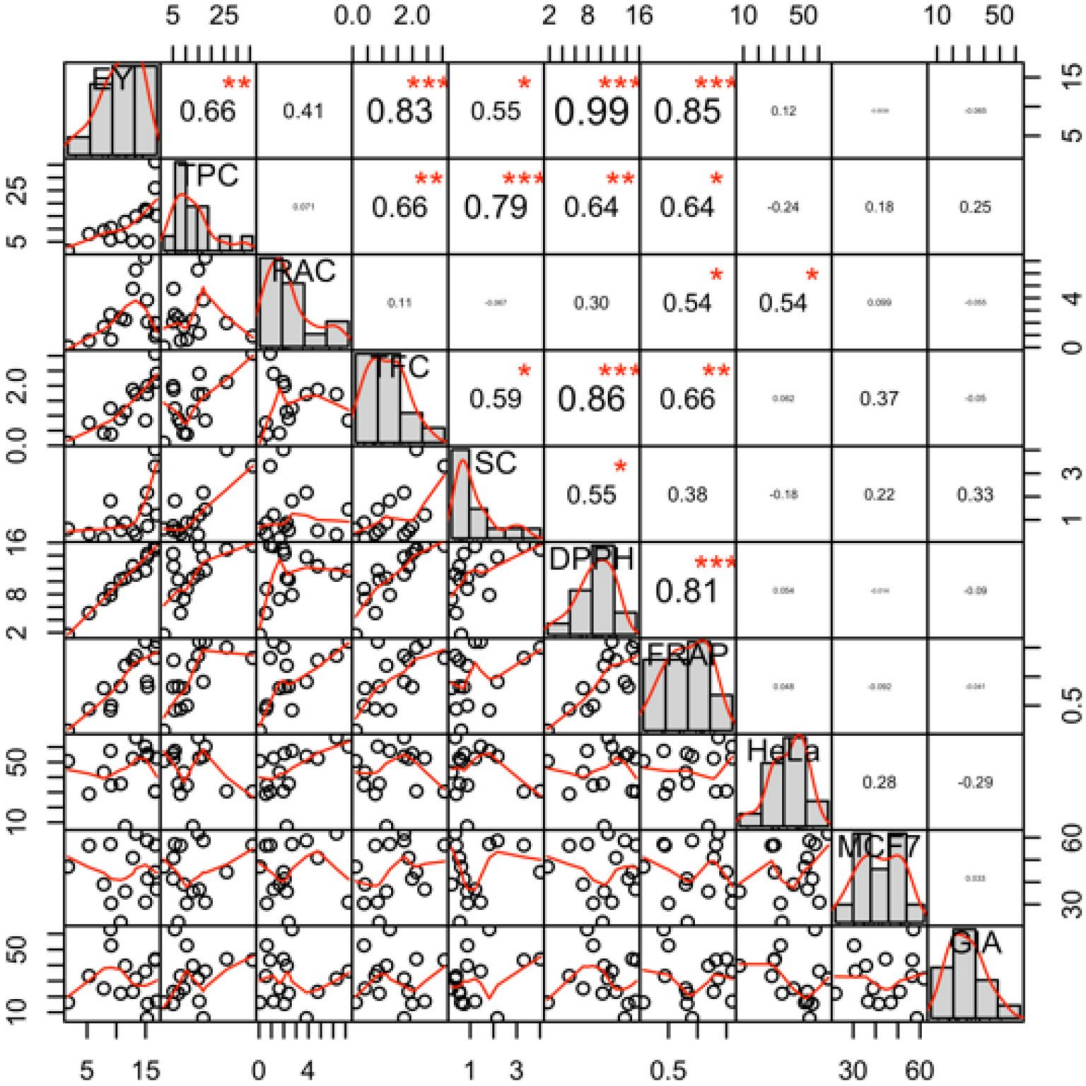
Correlation matrix chart of phytochemical content and pharmacological parameters. The upper panels show the Pearson’s correlation coefficients while the lower panels report the scatter plots. *, **, *** indicates significance at p < 0.05, < 0.01, and 0.001, respectively. For an explanation of parameter symbols, see Tables 1 and 2.

Figure 1 shows the correlation matrix chart of phytochemical content and pharmacological activities from the ethanol extract of *O. aristatus* studied genotypes. Extraction yield showed a significantly positive correlation with TPC, TFC, SC, DPPH, and FRAP. TPC recorded considerably correlated with TFC, SC, DPPH, and FRAP. TFC significantly correlated with SC, DPPH, and FRAP. SC associated considerably with antioxidant activity (DPPH assay). Furthermore, two tests of antioxidant activities were used in this study, DPPH, and FRAP, which showed a strong correlation. Significant associations were also found between RAC & FRAP and RAC & HeLa. MCF-7 and GIA showed the lowest correlation with other parameter studied of phytochemical components, extraction yield, and biological activities.

**Figure 2:**
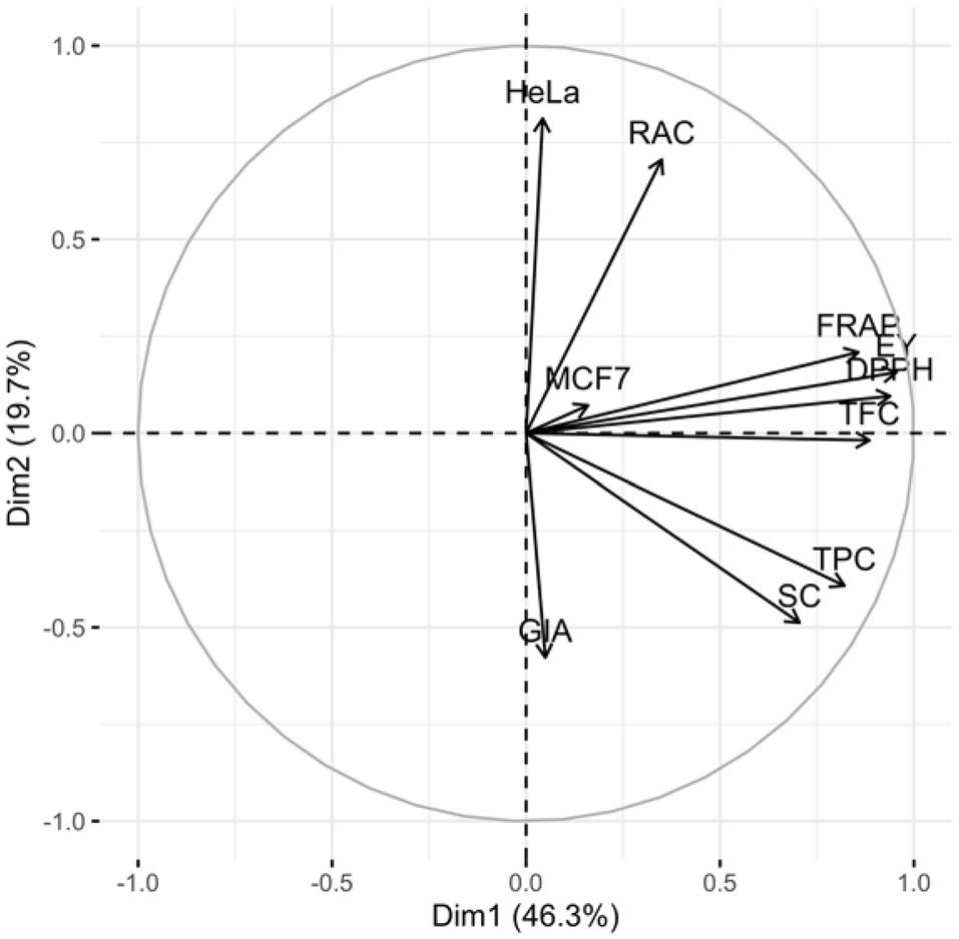
Loading plot of phytochemical content and pharmacological parameters. For an explanation of parameter symbols, see Tables 1 and 2.

**Figure 3:**
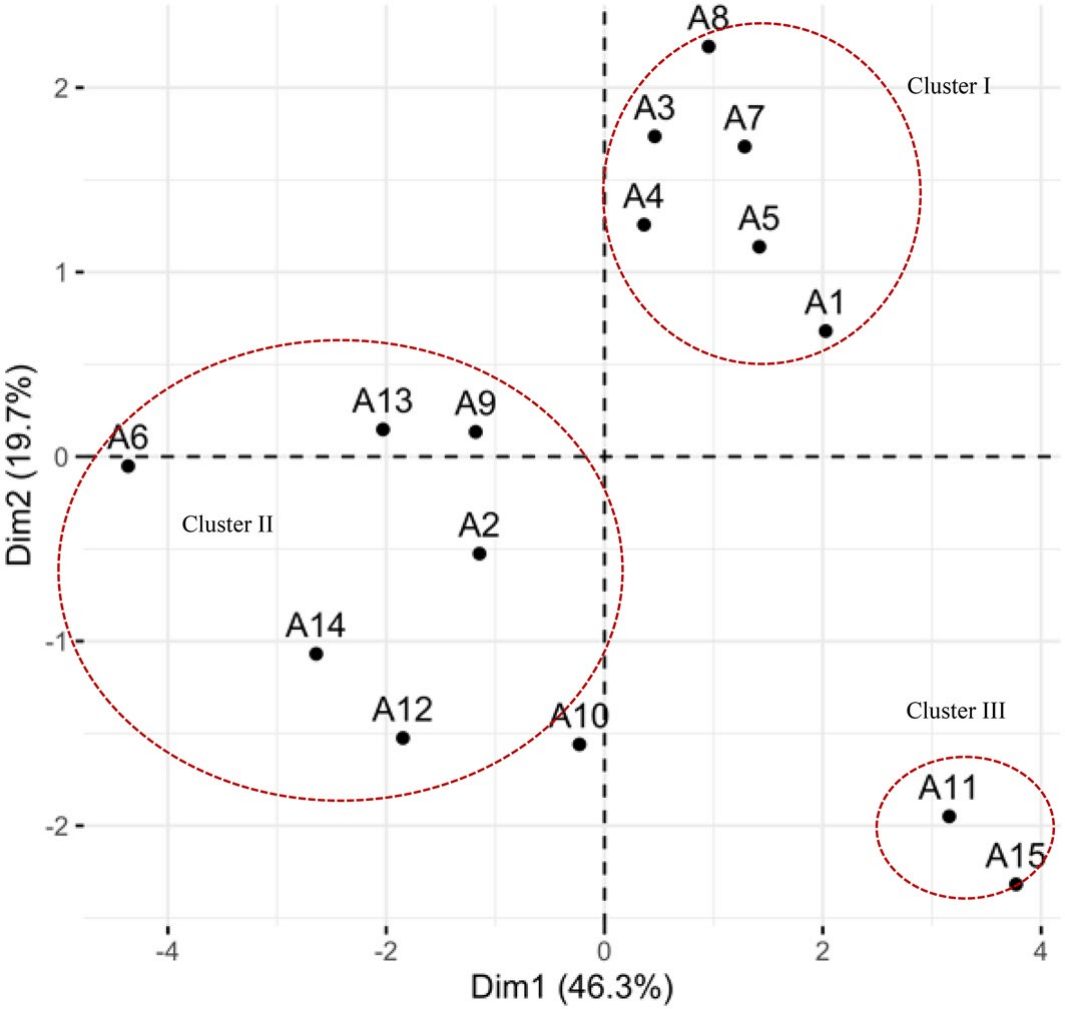
Score plot of 15 *O. aristatus* genotypes based on phytochemical content and pharmacological activities. For an explanation of parameter symbols, see Tables 1 and 2.

The principal component analysis was performed for phytochemical contents and pharmacological activities to evaluate any promising group within the *O. aristatus* genotypes. Result PCA analysis in the first five principal components presented in Table 3. The first five principal components resulted in 93.31% of the cumulative percent of the variance. PC1 and PC2 explained 46.34% and 19.67% of variance with eigenvalue 4.63 and 1.97, respectively. PC3 to PC5 having smaller eigenvalue, thus these not significantly explained the variance in datasets (27.31%). Thus, PC1 and PC2 selected for explaining the variance. Figure 2 shows the loading plot of phytochemical content and pharmacological parameters. EY, TPC, TFC, SC, DPPH, and FRAP parameters had the significant effects on the PC1. Meanwhile, RAC and HeLa parameters had strong influences on the PC2. Biplot analysis of phytochemicals and biological properties created from the comparison of component one-two PCs revealed three distinct clusters of *O. aristatus* genotypes (Fig. 3). Four groups resulted, as shown in Fig. 1. Cluster I comprised of six genotypes viz. A1, A3, A4, A5, A7, and A8 with characterized by high rosmarinic acid and cytotoxic (HeLa & MCF-7) activity, intermediary for other phytochemical and antioxidant activity, and lowly GIA. Cluster II has composed seven genotypes: A2, A6, A9, A10, A12, A13, and A14. These genotypes characterized by low phytochemical content and biological activities, except high for GIA. Cluster III composed two genotypes (A11 and A15) and characterized by high total phenolic content, total flavonoid content, sinensetin content, extraction yield, and antioxidant activity.

**Table 3:** First five principle components and its eigenvalue and percent variation explained for phytochemical content and pharmacological activities of *O. aristatus* genotype.

Parameters^a^	Dim. 1	Dim. 2	Dim. 3	Dim. 4	Dim. 5
EY	0.955	0.157	− 0.170	− 0.035	0.095
TPC	0.821	− 0.393	0.085	0.063	− 0.120
RAC	0.350	0.706	0.009	0.543	− 0.197
TFC	0.885	− 0.019	0.196	− 0.297	0.005
SC	0.705	− 0.488	0.250	0.040	0.257
DPPH	0.939	0.095	− 0.189	− 0.137	0.104
FRAP	0.857	0.209	− 0.301	0.156	− 0.219
HeLa	0.043	0.812	0.368	0.087	0.404
MCF7	0.160	0.072	0.933	− 0.121	− 0.272
GIA	0.050	− 0.578	0.184	0.730	0.122
Eigen value	4.63	1.97	1.30	0.99	0.44
Percent of variance	46.34	19.67	13.03	9.89	4.39
Cumulative percent of variance	46.34	66.01	79.04	88.92	93.31

^a^For an explanation of parameter symbols, see Table 1 and Table 2.

## Discussion

The phytochemical content and pharmacological effects of a medicinal plant species will differ depending on the geographical conditions where the plant grows^25,26^. Therefore, to select medicinal plants that have superior performance for the plant breeding programs, limited environmental conditions are needed^27,28^. In this study, *O. aristatus* collected from Indonesia was investigated for the characterization of phytochemicals contents and pharmacological activities and obtained promising elite genotypes without environmental impacts. The phytochemical and pharmacological distinction of *O. aristatus* has economic significance as well as being accommodating in the development of pharmaceutical industries and plant breeding programs for new varieties. Fifteen genotypes of *O. aristatus* were assessed for the quality of phytochemicals and pharmacological activities in all records of this discussion.

Phenolic is a group metabolite associated with several pharmacological activities; therefore, the content of phenolics (TPC) in the plant needs to be determined^29,30^. TPC ranged 1.48 mg GAE/g DW (A6) to 36.08 (A15) mg GAE/g DW (Table 1). TPC found in the leaves of *O. aristatus* is varied based on the different solvent used in the extraction process, different extraction methods, different extraction time and different environments the plant as well as the different of genotypes. Farhan et al.^31^, found the total phenolic content in the leaves as 230 mg GAE/g DW using methanol for extraction, Abdelwahab et al.^32^, reported TPC as 52.10 mg GAE/g DW from 60% methanol extract, and the lowest TPC content found is 20.03 mg GAE/g DW for extraction using 40% ethanol for 120 min at 65 ℃^33^. The solvent for extraction used in this study is 70% ethanol, so the TPC of the extract in this study compares with the reports of Chew et al.^33^. The difference is our experiment without additional heat and Chew et al. using 65 ℃. Based on the dried weight of the leaves, the TPC of the genotype A15 is higher than reported by Chew et al.^33^. Phenolic compounds in *O. aristatus* leaves are from flavonoid groups, tannins, and simple phenolics. Simple phenolic compounds include rosmarinic acid (RAC) that is also determined in this study (Table 1). RAC ranged 0.06 mg/g DW (A6) to 7.25 mg/g DW (A7). There is no correlation between TPC and RAC (Fig. 1). It means the phenolic extracted by 70% ethanol is not only rosmarinic acid. Genotype A15 and A7 could be used to *O. aristatus* varieties development for high phenolics productivity in plant breeding program.

The *O. aristatus* leaves have a lot of types of flavonoids but the concentration is low. Methoxy flavonoid is the known type of flavonoids in this leaves. Sinensetin, salvigenin and eupatorine are methoxy-flavonoid groups in *O. aristatus*. The total flavonoid and sinensetin contents of all extracts are shown in Table 1. The report of Abdelwahab et al.^32^, showed that 60% methanolic extract of *O. aristatus* leaves had 17.46 mg RE/g extract. This report is equal to 8.69 mg QE/g extract. Different from the report of Chew et al.^33^, who reported TFC in dried leaves of *O. aristatus* is about 1611.9 mg CE/100 g DW which equal to 16.78 mg QE/g DW. Our best TFC content found in A15 is only about 3.07 mg QE/g DW. The sinensetin content in the dried materials of *O. aristatus* leaves, the highest was found on A11, while the lowest on A8. There is correlation between SC and TFC (Fig. 1). It means that the sinensetin is major flavonoid in studied extract genotypes. On the other hand, it was reported that sinensetin content will decrease when extraction was performed using polar solvent such as ethanol 70%^34^. This result indicated that the genotype A15 and A11 could be developed through a breeding program to get the high flavonoid and sinensetin contents of *O*. *aristatus* varieties.

Recently, an antioxidant is of particular concern, which played a critical role in preventing several human illnesses^35^. Thus, getting plant genotypes as a source of high antioxidant activities is essential^36^. In this study, DPPH and FRAP assays were used to evaluate the antioxidant activities in ethanol extract of *O. aristatus* studied genotypes. DPPH assay was used to analyses the scavenging capacities of extract, while the reducing power was determined by FRAP assay^37^. In present work, the *O. aristatus* genotypes studied showed variation for DPPH (1.68 to 15.55 μmol TE/g DW) and FRAP (0.07 to 1.60 µmol TEAC/g DW) (Table 2). The highest antioxidant activities in DPPH and FRAP were found on genotype A15 and A7, respectively. In DPPH assay, our *O. aristatus* genotypes had lower antioxidant capacity compared with reported by Chew et al.^33^ which about 21.81 µmol TEAC/g DW. Scavenging activity of genotype A15 recorded is not significantly different from A1, while genotype A7 and A1 were found not different in reducing power activities. This result indicated that the genotype A1 could be used to develop as *O. aristatus* plant producing high antioxidant properties. Furthermore, antioxidant activities strong correlated with TPC and TFC indicates that the polyphenol (phenolic and flavonoid) in *O. aristatus* genotypes are the major metabolites for antioxidant activity. Similarly, Saidan et al.^38^ while evaluating the different genotypes, reported that the correlation between TPC and DPPH activity was about 0.90 and FRAP 0.78, while between TFC and DPPH was 0.73 and FRAP 0.71. Our results show that the rosmarinic acid content on *O. aristatus* leaves has a positive correlation with the DPPH activity and with FRAP activity of the extracts. The R^2^ of the correlation between RAC and DPPH activity (0.30) is lower than with FRAP activity (0.54) (Fig. 1). This result is agreed with the report of Ho et al.^39^ which concludes that radical scavenging of *O. aristatus* is related to the high content of rosmarinic acid. Since the R^2^ is not higher than 70%, it means other compounds also responsible for the antioxidant activity. It can be understood that besides phenolic and flavonoid responsible to the antioxidant activity of *O. aristatus*, diterpene, triterpene, and saponin groups are also responsible for antioxidant activity^38,40^.

Cytotoxic activity of 15 *O. aristatus* genotypes against HeLa and MCF-7 cell lines is summarized in Table 2. In this work, the *O. aristatus* genotypes studied showed cytotoxic variation for HeLa (7.33 to 66.25%) and MCF-7 (21.96 to 61.79%). In both cell lines, genotype A8 recorded the highest in cytotoxic activity. The previous report on the cytotoxic study of *O. aristatus* leaves extracts concludes that this extract is toxic against breast cancer (MCF-7, 53.4% inhibition) and colon cancer (HCT116, 43.7% inhibition) cell lines^38^. Our results show better cytotoxicity compared with the previous report of Saidan et al.^38^. *O. aristatus* extract has also been reported to have cytotoxic activity against prostate cancer (DU145) with no harmful effect against normal fibroblast cell of HSFF 1184^34^. Saidan et al.^38^ conclude that the cytotoxic activity of *O. aristatus* extract had a positive correlation with TPC and TFC. This phenomenon is not found in our results. There is no correlation between TPC and TFC with cytotoxic activity against MCF-7 and HeLa. However, MCF-7 presented weakly positive correlation with TFC and SC (Fig. 1). These indicate that the flavonoid and sinensetin compounds are responsible for cytotoxic activity in *O. aristatus* extract. Previous work has shown that methoxylated flavonoids (eupatorine and sinensetin) have contributed greatly to the cytotoxic activity of *O. aristatus* extract^41,42^. Our results also show that there is a correlation between cytotoxic activity and rosmarinic acid, which in line with the previous report of Suhaimi et al.^34^. Suhaimi et al.^34^ said that the highest content of rosmarinic acid showed an anti-proliferative effect against prostate cancer. Results indicated that the genotype A8 could be developed through a breeding program to get the high anticancer capacities of *O. aristatus* varieties.

The *O. aristatus* genotype namely A2 (62.84%) showed high *α-*glucosidase inhibitory activity. Sinensetin and 5-hydroxy-6, 7, 3′, 4′-tetramethoxyflavone together with diterpenes and triterpenes are reported responsible for *α-*glucosidase inhibition activity in *O. aristatus*^40^. Our results show that there is no correlation between *α-*glucosidase inhibition activity with TPC, TFC, RAC and also SC. As reported by Yuliana et al.^40^, other components such as diterpenes and triterpenoids are responsible for *α-*glucosidase inhibitory activity.

Multivariate analysis, namely chemometric, is the statistical procedure to enhance the understanding of metabolite information and to associate quality traits to data of analytical instrument. It has been used to study in the correlation of the phytochemical component with pharmacological activities in the plant^20,28,43^. In this study, we used two chemometric methods namely correlation and principal component analysis (PCA) to evaluate the phytochemical and pharmacological properties of the *O. aristatus*. Antioxidant activity (DPPH & FRAP) found a significantly positive correlation with total phenolic and total flavonoid contents (Fig. 1). These indicate that they are significant selections parameters in cultivating the *O. aristatus*. Furthermore, these results indicated that the polyphenols are the main compounds for antioxidant capacities in *O. aristatus*. The results are in line with previous reports in *O. aristatus* and other different crops^38,44,45^. Besides, several reports shows association between polyphenol contents and antioxidant properties, but no one highlighted the mechanism of the association between polyphenol compounds and antioxidants in *O. aristatus*. This work showed a strong correlation between rosmarinic acid & FRAP assay and sinensetin & DPPH assay. Results indicated that the antioxidant mechanism of rosmarinic acid from *O. aristatus* as reducing power while free radical scavenging mechanism found in the sinensetin compound. Interestingly, in PCA using phytochemical contents and pharmacological parameters, it was resulted three clusters. Two *O. aristatus* genotypes viz. A11 and A15 had high TPC, TFC, SC, EY, DPPH, and FRAP, indicating a correlation of high polyphenol in these genotypes with antioxidant activities and extraction yields. Six *O. aristatus* genotypes viz. A1, A3, A4, A5, A7, and A8 had high rosmarinic acid and cytotoxic activities (HeLa & MCF-7), indicating the correlation of high rosmarinic acid content in these genotypes with cytotoxic properties. In the future, combining with anticancer activities such as A8 genotype with high phytochemical contents such as A15 genotype will be the main goal for new varieties development as anticancer sources with valuable phytochemical-rich in *O. aristatus*.

Fifteen *O. aristatus* genotypes had phytochemical and biological activities diversity. Genotypes of A11 and A15 produced high total phenolic and flavonoid contents, genotype A7 produced high rosmarinic acid content, and genotype A11 produced high sinensetin content. The genotype A1 and A7 had the best antioxidant activity against FRAP, while genotype A15 had the highest antioxidant activity against DPPH assay. The genotype of A2 had the best cytotoxic and *α-*glucosidase inhibition activities. The selected genotypes with different purposes could be developed in plant breeding.

## Methods

### Plant materials and sample preparation

Plant materials consisted of 15 different *O. aristatus* genotypes, belonging to dissimilar plant height (short, medium and tall), anthocyanin coloration on stem (absent or very weak, weak, medium and strong), leaf shape (narrow elliptic, medium elliptic, and medium ovate), flower colour (white and purple), and maturity group (early and late) (Table 4). Fifteen types of *O. aristatus* were cultivated in the experimental fields of Tropical Biopharmaca Research Center, Bogor Agricultural University, West Java, Indonesia (6°32′25.47″ N, 106°42′53.22″ E, 142.60 m above the sea level) with the randomized complete block design in three replications. For the evaluation of phytochemicals and biological activities, leaves were harvested at three months after planting. After harvesting, the leaves of each genotype were dried in the oven (50 °C). The powder (100 mesh) samples were extracted in 70% ethanol using the maceration method described by Abdullah et al.^46^, with slight modification. Briefly, 15 g leaves samples were taken and their extraction was performed in 150 ml ethanol for 24 h at 150 rpm rotation speed (Eyela multi shaker MMS-Germany) and room temperature. The solution was filtered with Whatman no. 4. Then, rotary evaporator was applied to evaporate the extracts. To calculate the extraction yield, the extracts were gathered and weighed. Resulted extracts were kept at 4 °C until further usage.

**Table 4:** Detailed description of the *O. aristatus* genotypes for plant, leaves and flower characters.

Genotypes	Plant height	Anthocyanin coloration on stem	Leaf shape	Flower colour	Maturity group
A1	Medium	Medium	Narrow elliptic	White	Early
A2	Short	Weak	Medium elliptic	Purple	Early
A3	Medium	Absent or very weak	Medium ovate	White	Early
A4	Medium	Weak	Medium elliptic	White	Late
A5	Medium	Strong	Medium elliptic	White	Late
A6	Tall	Strong	Narrow elliptic	White	Late
A7	Medium	Weak	Medium elliptic	White	Early
A8	Medium	Medium	Narrow elliptic	White	Late
A9	Medium	Medium	Narrow elliptic	White	Late
A10	Short	Strong	Medium elliptic	White	Late
A11	Medium	Strong	Medium ovate	White	Late
A12	Medium	Strong	Medium elliptic	White	Late
A13	Medium	Medium	Medium elliptic	White	Late
A14	Tall	Absent or very weak	Medium elliptic	White	Late
A15	Medium	Absent or very weak	Narrow elliptic	White	Late

Characterization for plant height (≤ 30 cm: short, 30–60 cm: medium, ≥ 60 cm: tall); Anthocyanin coloration on stem, leaf shape and flower colour followed from UPOV^57^; days took to flower defined for maturity group ((≤ 34 day: early; ≥ 35 cm: late).

### Determination of total phenolic and flavonoid content

Total phenolic and flavonoid content were determined using spectrophotometrically suggested by Khumaida et al.^47^. Total phenolic content in each extract was determined using the Folin-Ciocalteu reagent and gallic acid was used as an external standard. About 10 μl extract sample was mixed with distilled water (160 μl), 10% Folin-Ciocalteu reagent (10 μl), and 10% Na_2_CO_3_ (20 μl). After incubation at room temperature for 30 min, the absorbance was measured using the microplate reader (Epoch BioTek, USA) at 750 nm. The total phenolic content was expressed as gallic acid equivalent (mg GAE/ g DW). Total flavonoid content was measured using aluminium chloride reagent and quercetin was used as an external standard. In a 96-well microplate, a 10 μl extract sample was mixed with methanol (60 μl), 10 μl of 10% aluminium chloride, 10 μl of 1 M potassium acetate, and 120 μl of distilled water. Finally, after incubation at room temperature for 30 min, the absorbance was measured using the microplate reader (Epoch BioTek, USA) at 415 nm. Results expressed as quercetin equivalent (mg QE/ g DW).

### Determination of sinensetin and rosmarinic acid content

Sinensetin and rosmarinic acid contents in ethanol extract of *O. aristatus* genotypes were measured by high performance liquid chromatography (HPLC) according to a previously reported method^48^ with modification. Standard solutions of rosmarinic acid and sinensetin, obtained from ChromaDex, were prepared separately in methanol at a range concentration of 1–100 μg/ml and 0.3–24 μg/ml, respectively. The Shimadzu HPLC (Shimadzu, Kyoto, Japan) was used to perform the analysis which was equipped with degassing unit (DGU-20A5R), pump (LC-20AB), autosampler (SIL-20A HT), column oven, (CTO-20AC), and UV–Vis detector (SPD-20A). The column used Shim-Pack VP ODS C18 Shimadzu (75 × 4.6 mm i.d., 3 μm pore size). The 0.1% formic acid in aqueous (A) and 0.1% formic acid in acetonitrile (B) were used as a mobile phase of elution. The gradient elution system used as follow: 0–7 min for 2–20% B, 7–10 min for 20–30% B, 10–18 min for 30–50% B, 18–20.5 min for 50–98% B, 20.5–23 min for 98% B, 23–26 min for 98–2% B, and the system was then balanced until 35 min before the next injection. Each genotype sample, the volume of injection was 10 µl, was analysed at 1 ml/min for the mobile phase flow rate and 320 nm for detector wavelength at 30 °C for 35 min. Results of rosmarinic acid and sinensetin content expressed as mg/g DW based on the calibration curve of rosmarinic acid and sinensetin standard, respectively.

### Determination of antioxidant activity

Two *in-vitro* methods were applied to analyse antioxidant activities. DPPH and FRAP methods were used to evaluate the free radical scavenging and reducing power activities, respectively. Antioxidant activity was expressed as Trolox Equivalent with Trolox (vitamin E analogues) as an antioxidant standard. Trolox was frequently used as an α-tocopherol model compound^49^. Furthermore, Trolox is used to express the antioxidant capacities of food, chemical compounds and biological matrices, as a standard antioxidant compound in terms of Trolox equivalent antioxidant capacity (TEACH)^50^. For this reason, Trolox can be considered as the proper counterpart of Vitamin E to examine its actions in aqueous radical environments on its own and in combination with other antioxidant compounds.

For 2,2-diphenyl picrylhydrazyl (DPPH) method proposed by Nurcholis et al.^51^, with modification. Briefly, 100 µl *O. aristatus* ethanol extract was added to 100 µl of 125 µM DPPH in methanol at the 96-well microplate (Costar-USA). After incubation in darkroom temperature for 30 min, the absorbance was measured using the microplate reader (BMG Labtech, Germany) at 517 nm. Results expressed in µmol Trolox Equivalent (TE/g DW).

FRAP was determined according to the assay of Benzie and Strain^52^ with modification. FRAP reagent was prepared by mixing 300 mM acetate buffer pH 3.6, 10 mM TPTZ in 40 mmol HCl, and 20 mmol FeCl_3_ in the ratio of 10:1:1 (v/v/v). One hundred µl *O. aristatus* ethanol extract was added to 300 µl of FRAP reagent in the 96-well microplate (Costar-USA). After incubation at 37 °C, the absorbance was measured using the microplate reader (BMG Labtech, Germany) at 593 nm. Antioxidant activity was expressed as µmol Trolox Equivalent (TE/g DW).

### Determination of cytotoxic activity

Cytotoxic activity was measured by the hemocytometer method according to previous reported by Fitria et al.^53^. MCF-7 and HeLa cancer cell lines, obtained from Veterinary Medicine Faculty, IPB University, were used cytotoxic evaluation. Cell lines were grown in medium which consist of 850 μl Dulbecco's minimum Eagle's medium (Gibco, Rockville, MD, USA), 30 μl fetal bovine serum (10%; Sigma–Aldrich, St. Louis, MO, USA), and 10 μl fungizone (Gibco, Rockville, MD, USA). In brief, 50 μl cell line were exposed to a sample of 100 μg/ml. Also included, the control group was an untreated cell line. The cell incubated at 37 °C in 5% CO_2_ for 3 days. After incubation, 100 μl cells were added to 5 μl trypan blue. After homogenized, 10 μl cells were placed in a hemocytometer for calculation number of cells. The cytotoxic activity was determined based on the treated cell and untreated cells in percentage inhibition.

### Determination of α-glucosidase inhibitory activity

The α-glucosidase inhibitory activity in *O. aristatus* genotype was performed according to the assay previous reported^54^. The extract sample (10 μl) of concentration 1000 μg/ml was a mixed with 0.1 M phosphate buffer pH 7.0 (50 μl), 0.5 mM pNPG (25 μl), and 0.2 unit/ml α-glucosidase solution (25 μl). Then, the mixture incubated at 37 °C for 30 min. Finally, the reaction was stopped by 200 mM Na_2_CO_3_ (100 μl) and the absorbance was measured using the microplate reader (Epoch Biotech, USA). Results expressed as percentage inhibition of α-glucosidase activity. The percentage of α-glucosidase inhibition (GIA) was calculated using formula (1):

1 $$GIA (\%)=\frac{{A}_{control }{- A}_{sample}}{{A}_{control }}\times 100$$

where the control reaction absorbance (with all reagents but one of its extract) is A_control_ and the extract absorbance tested in the reaction mixture is A_sample_.

### Statistical analysis

Data were expressed as the mean ± SD from three replicates. ANOVA was analysed using SPSS version 25. R was used for multivariate analyses using correlation and principal component analysis. The Pearson correlation coefficients between phytochemical and pharmacological parameters were generated using PerformanceAnalytics packages in R^55^. The FactoMineR packages in R was used to create PCA analysis using data matrix of phytochemical and pharmacological variables^56^.

## Abbreviations

DPPH: 2,2-Diphenyl picrylhydrazyl
FRAP: Ferric reducing antioxidant power
TE: Trolox equivalent
DW: Dry weight
GAE: Gallic acid equivalent
QE: Quercetin equivalent
TPC: Total phenolic content
TFC: Total flavonoid content
RAC: Rosmarinic acid content
SC: Sinensetin content
EY: Extraction yield
HeLa: Anticancer activity of HeLa cancer cell line
MCF-7: Anticancer activity of MCF-7 cancer cell line
GIA: α-Glucosidase inhibitory activity
PCA: Principle component analysis
